# Hydrogel-based dressings in the treatment of partial thickness experimentally induced burn wounds in rats

**DOI:** 10.1590/acb370401

**Published:** 2022-07-01

**Authors:** Milton Junior Cândido Bernardes, Randys Caldeira Gonçalves, Carolyna de Sousa Carvalho, Luciana Martins Rosa, Amanda Peixoto Ferreira, Marielle Sousa Vilela, Marina Clare Vinaud, Hélio Galdino, Ruy de Souza Lino

**Affiliations:** 1 PhD. Universidade Federal de Goiás – Tropical Pathology and Public Health Institute – Postgraduation Program in HostParasite Relationship – Goiânia (GO), Brazil.; 2 MSc. Universidade Federal de Goiás – Tropical Pathology and Public Health Institute – Postgraduation Program in HostParasite Relationship – Goiânia (GO), Brazil.; 3 Graduate student. Universidade Federal de Goiás – Goiânia (GO), Brazil.; 4 Nurse. Universidade Federal de Goiás – Nursing School – Goiânia (GO), Brazil.; 5 PhD. Universidade Federal de Goiás – Tropical Pathology and Public Health Institute – Biosciences Department – Goiânia (GO), Brazil.; 6 PhD. Universidade Federal de Goiás – Nursing School – Goiânia (GO), Brazil.

**Keywords:** Burns, Wound Healing, Hydrogels, Rats

## Abstract

**Purpose:**

To compare four commercially available hydrogel formulations in the healing of partial thickness burns experimentally induced in rats.

**Methods:**

Wistar rats were used, and after the burn wound induction they were divided into the following treatment groups: G1) NaCl 0.9%; G2) 1% silver sulfadiazine; G3) Debrigel™; G4) Safgel™; G5) Dersani™; G6) Solosite™. The animals were followed during seven, 14 and 30 days after the injury induction. Morphometric, macroscopic and microscopic evaluations were performed.

**Results:**

The treatment with Dersani™ induced better results during the inflammatory and proliferative phases of the healing process (p<0.05). The animals treated with Safgel™ presented better scaring in the remodeling phase (p<0.05), and the treatment with Dersani™ and Solosite™ induced greater wound closure (p<0.05).

**Conclusions:**

The hydrogel-based dressings presented beneficial outcomes in the healing of burn wounds experimentally induced in rats due to their ability in maintain the humidity of the wound, in removing the exudate, in promoting cell migration and collagen production during the different phases of the healing process.

## Introduction

Burn injuries are a significant cause of mortality and morbidity worldwide, being the cause of 265.000 deaths and 11.271 indefinitely disabled people^1^. About 90% of burn injuries occur in Asia, Africa, and the Americas. Among the causal factors are culinary practices, flammable clothing, and flammable fuels. Children under the age of 5 are more vulnerable to burn injuries. Individuals who are victims of burns must deal with deficiencies throughout their lives and deformities caused by tissue contractures, mainly due to the lack of adequate and timely resources for treatment^1^^-^ 3.

In the most affected geographic areas, access to adequate care for burns is limited, both due to the lack of health infrastructure and the costs of available treatments. A cost analysis of a hospital in Turkey revealed that the average cost for treating flammable product burn victims was US$ 368 per 1% of the burned area surface. Moreover, although intensive care unit care has been the main cost factor (27%), dressings constituted 15% of the cost of care^3^^,^ 4.

Partial thickness burns require frequent changes of antimicrobial dressings. The absence of dressings leads to increase in the susceptibility to infection, desiccation, additional trauma, and delayed re-epithelialization^5^^-^ 7. The treatment of choice for superficial partial-thickness burns is the topic use of silver sulfadiazine. Despite being the treatment of choice, it has limitations such as adherence to the wound bed, requiring greater friction of the wound bed, so that it can be completely removed at the moment of dressing changes^8^^-^ 11. However, a variety of new products for this purpose are emerging. Hydrogels are options for the treatment of burns, being composed of a system formed by water and a hydrophilic polymer, which due to its three-dimensional structure can perform ion and gas exchange and favor the healing process. The properties of hydrogels favor the ideal humid environment for healing, in addition to being more practical, easy to apply, remove and atraumatic, being, therefore, widely used in clinical practice^9^^,^ 11 ^-^ 16.

In experimental studies, low-cost alternatives for the treatment of burns have been explored. The hydrogel is easy to handle, stable and has the possibility of being manipulated together with various compounds and natural substances. Among the compounds that can be manipulated with the hydrogel are sodium and/or calcium alginate. Although hydrogels with alginate have shown benefits in terms of wound healing, their effects are not completely understood, regarding its microscopic and macroscopic effects on the healing process of burn wounds^17^^-^ 19.

Regarding all the benefits described in a hydrogel-based dressing for burn wounds, we believe that this kind of wound would benefit and present better healing process with it. Therefore, the aim of this study was to evaluate the macroscopic and microscopic evolution of partial thickness burns in rats, submitted to treatment with hydrogel dressings with calcium and/or sodium alginate in comparison to the standard treatment of silver sulfadiazine.

## Methods

This study was carried out at the Tropical Pathology and Public Health Institute, Universidade Federal de Goiás (UFG), in collaboration with the company CuraCenter Centro Clínico e Tratamento de Feridas LTDA. The study was approved by the Ethics Committee on the Use of Animals (CEUA-UFG) – Protocol No. 084/17.

### Experimental animals

This is an experimental study that used 90 female Wistar Hannover (*Rattus norvegicus albinus*) rats, weighing approximately 200-250 g, about 8 weeks old, from the Central Animal Facility of UFG.

Three rats were kept per cage. All the cages used were made of polypropylene, lined with wood shavings, and the bedding was changed twice a week. All the animals received water and commercial autoclaved food *ad libitum*. Luminosity and temperature were controlled, and noise intensity and relative air humidity were those of the general environment.

### Experimental groups and products used

For the experimental procedure, the animals were randomly distributed into six groups:

- G1: control with saline (NaCl 0.9%);
- G2: control treated with 1% silver sulfadiazine;
- G3: test treated with Debrigel™;
- G4: test treated with Safgel™;
- G5: test treated with Dersani Hydrogel™;
- G6: test treated with Solosite™.

All groups were monitored daily and euthanized at seven, 14 and 30 days after burn induction (DAI), using five rats per experimental day, the minimum number required in order to allow the statistical analysis.

### Products composition

- Silver sulfadiazine: cetostearyl alcohol, cetomacrogol 1,000, liquid petrolatum, propylene glycol, methylparaben, propylparaben, butylhydroxytoluene, and purified water. Manufacturer Prati-Donaduzzi Brasil, lot 16A273.

### Hydrogels

- Debrigel™: calcium sodium alginate, sodium carboxymethyl cellulose, propylene glycol, boric acid, idantoin, potassium sorbate and triethanolamine. Manufacturer Helianto Farmacêutica LTDA, lot 145730;
- Safgel™: hydrocolloids (carboxymethylcellulose and carbomer), sodium and calcium alginate, propylene glycol, hydroxypropylparaben, hydroxymethylparaben, imidazolidynyl, aminomethylpropanol, and ultra-purified water. Manufacturer ConvaTec Incorp Limited, lot 43265C.
- Dersani Hidrogel™: capric and caprylic acid triglycerides; clarified sunflower oil, lecithin, retinol palmitate, tocopheryl acetate, alpha-tocopherol, sodium alginate, essential fatty acids, vitamins A and E, propylene glycol, disodium adedate, sodium benzoate, carbomer, sodium hydroxide, and purified water. Manufacturer Daudt Oliveira LTDA, lot 3069N;
- Solosite™: purified water, glycerin, sodium carboxymethyl cellulose, allantoin, benzyl alcohol, methylparaben, propylparaben, carboxymethyl cellulose (CMC) modified polymer, propylene glycol, and water. Manufacturer Smith & Nephew Medical Limited, lot 17698.

### Burn wound induction procedure

On day 0, the animals were previously weighed and anesthetized by intraperitoneal administration of an anesthetic solution of 10% ketamine (União Química Farmacêutica Nacional S/A, Brazil) and 2% xylazine (Sespo Indústria e Comércio LTDA, Brazil). A volume of 0.1 mL/100 g of animal weight was injected per animal. After application of the anesthetic solution, shaving the dorsal region of the animal was performed, with subsequent antisepsis of the area to be burned, using sterile gauze soaked in 70% alcohol solution. To perform the lesion, the animal was placed inside a polyvinylchloride (PVC) plastic cylinder, with an opening of 2 × 2 cm^2^ and sealed ends. Then, a partial-thickness thermal lesion was performed by immersing the animal’s back area exposed to boiling water at 95°C for seven seconds. This time of exposure to boiling water is capable of causing injury until the deep partial thickness of dermis^20^^-^ 22. The resulting burned area corresponded to 12% of the animal’s body surface.

### Post-burn period

In the post-burn period, two animals were kept with occlusive dressings per cage, to maintain better comfort and avoid opening the dressings, thus minimizing the risk of possible trauma and contamination. The animals received analgesic medication: Tramal (Grünenthal do Brasil Farmacêutica LTDA) to reduce pain, diluted in the drinking fountain, during the first seven days, after the induction of the lesion. Diet remained *ad libitum*.

### Debridement

On the second day after the induction of lesions, the animals were previously weighed and anesthetized, using the same protocol for performing the burns, and underwent surgical debridement (tangential excision), according to the International Symposium on Biomedical Imaging^23^. To perform this procedure, a scalpel and scissors were used to remove the necrosis, and the skin was gently detached, preserving the subcutaneous muscle of the panniculus carnosus.

### Dressings

The animals in the control groups (G1 – control with NaCl 0.9% –; and G2 – control treated with 1% silver sulfadiazine) received daily occlusive and sterilized dressings. The animals in the silver sulfadiazine group received a uniform and thin layer of the product (1 mm), sufficient to cover the wound bed^24^. The dressings of the animals treated with the products Debrigel™, Safgel™, Dersani Hidrogel™ and Solosite™ were evaluated daily and had the dressings changed every two days, taking into account the specifications of the manufacturers. The secondary dressings were applied on top of the primary ones. Conditions such as clinical aspects, dirt, and humidity were checked daily. The secondary dressing was made of sterile gauze and a calico cloth.

### Euthanasia

The animals were euthanized at the end of each experimental day (seven, 14, and 30 days). The euthanasia was performed using a carbon dioxide (CO_2_) flow chamber.

### Morphometric evaluation

For morphometric analysis of wound contraction, the lesions were photographed using a digital camera attached to a tripod, at a constant distance of 11 cm. The images were recorded on day 0 (performing the burn) and at the end of each experimental day.

The delimitation of the burn area was performed using the ImageJ software (National Institutes of Health, United States of America). To determine the degree of contraction of the burn area, the following mathematical equation adapted from Moraes et al.^20^ was used (Eq. 1):

Relative wound contraction (%) = [(initial injured area - contracted injured area) / initial injured area] × 100 (1)

### Macroscopic evaluation

On the established experimental days, the phases of the healing process (inflammatory, proliferative and remodeling) were macroscopically analyzed. The presence of the following parameters was evaluated: necrosis/crust, granulation tissue and re-epithelialization, identified in a semi-quantitative way, according to the following criteria:

- Absent (score 0);
- Discreet (score 1 – up to 25% of compromised area);
- Moderate (score 2 – between 26 and 50% of compromised area);
- Severe (score 3 – above 50% of compromised area)^21^.

### Microscopic evaluation

The microscopic evaluation was performed using fragments of the wounds removed by means of biopsy and fixed in 10% buffered formaldehyde (pH 7.2). Subsequently, this material was processed through embedding in paraffin. The paraffin blocks were placed in a microtome (Leica RM2255), and serial sections of the material were obtained (4 μm) and placed on glass slides. The slides were stained using the hematoxylin and eosin (H&E) and picrosirius red (PS) techniques.

The general pathological processes were analyzed in the slides stained by the H&E using a binocular microscope (Leica DM750), coupled to a camera (Leica ICC50 HD) in order to record the images. The presence of the following parameters was observed: necrosis/crust, hemorrhage, fibrin, polymorphonuclear infiltrate (PMN), mononuclear infiltrate (MN), angiogenesis and fibroblasts, in which the entire length of the slide was evaluated. At 30 days after the injury induction (DAI), wound closure was analyzed. These parameters were identified in a semi-quantitative manner, following the criteria of Fantinati et al.^21^.

The collagen quantification was performed in the slides stained with PS, observed under a binocular microscope (Zeiss Axiostar Plus) and recorded with a digital camera (Sony Alpha Nex-3). Collagen fibers were evaluated under polarized light, and the entire length of the slides visualized and photographed. For this analysis, the ImageJ software (National Institutes of Health, United States of America) was used.

### Statistical analysis

Statistical analysis was performed using the SigmaStat 2.3 software. All variables were tested for normal distribution and homogeneous variance. For the morphometric analysis, the parametric analysis of variance (ANOVA) test and Tukey’s post-test were used. For macroscopic and microscopic analyses, the non-parametric Kruskal-Wallis test and Dunn’s post-test were used. Fisher’s exact test was used to assess the presence of microorganisms and analysis of wound closure. The differences were considered significant when p<0.05.

## Results

There were no animal losses during the experimental period of this study.

### Morphometric analysis

The degree of wound contraction was significantly higher at 14 DAI when the wounds were treated with Safgel™ in comparison to the control group. The other treatments did not interfere in the degree of wound contraction (Table 1).

**Table 1:** Morphometric analysis of the contraction degree of partial thickness burn wounds experimentally induced inWistar rats treated with calcium and/or sodium alginate hydrogel-based dressings#.

DAI	G1 (n=15) Medium ± SD	G2 (n=15) Medium ± SD	G3 (n=15) Medium ± SD	G4 (n=15) Medium ± SD	G5 (n=15) Medium ± SD	G6 (n=15) Medium ± SD	p-value	Tukey
14	24.9±12.8	47.9±22.8	49.1±17.2	61.5±14.8	35.9±16.8	44.7±11.8	0.02*	G4>G1
30	91.1±3.7	86.4±3.1	85.7±4.4	86.1±6.9	92.6±10.7	91.4±10.3	0.32	

DAI: days after injury induction; n: number of animals per group; G1: control group with animals treated with NaCl 0.9%; G2: animals treated with 1% silver sulfadiazine; G3: animals treated with Debrigel™; G4: animals treated with Safgel™; G5: animals treated with Dersani Hydrogel™; G6: animals treated with Solosite™; SD: standard deviation;

* statistical difference;

# statistical analysis – analysis of variance and Tukey’s post-test.

### Macroscopic analysis

**Figure 1:**
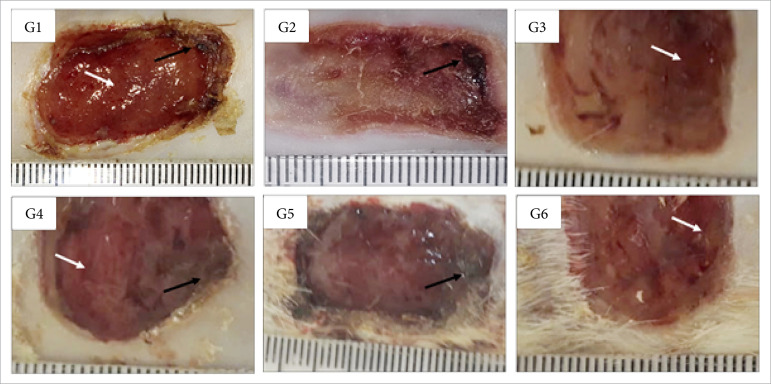
Macroscopic aspects of partial thickness burn wounds experimentally induced in Wistar ratsseven days after the injury induction. Scale in mm.

In the inflammatory phase of the healing process (7 DAI), there was significantly less necrosis/crust in the groups treated with Debrigel™ and Solosite™ than the one observed in the control group, treated with NaCl 0.9%, and to the group treated with Dersani™ (p<0.05). Granulation tissue was more detected in the group treated with Safgel™ in comparison to the treatments with 1% silver sulfadiazine, Debrigel™ and Dersani™ (p<0.05). The Solosite™ treatment also induced greater granulation tissue at this period in comparison to treatments with 1% silver sulfadiazine and Dersani™ (p<0.05) (Table 2; Fig. 1).

**Figure 2:**
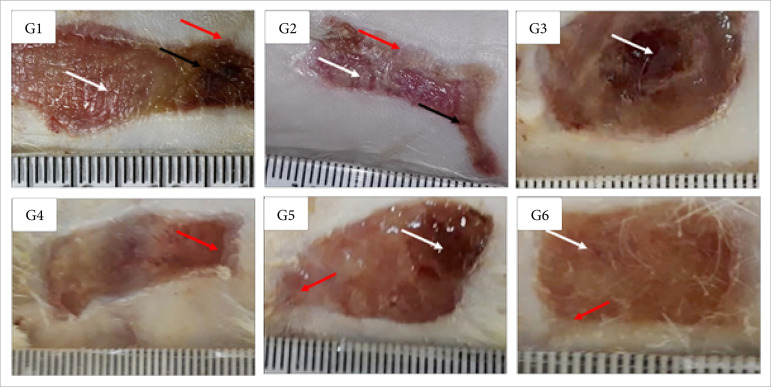
Macroscopic aspects of partial thickness burn wounds experimentally induced in Wistar rats 14 daysafter the injury induction. Scale in mm.

In the proliferative phase (14 DAI), there was greater necrosis/crust in the control group treated with NaCl 0.9% and the group treated with 1% silver sulfadiazine than the observed in the other treatments (p<0.05), while there was greater re-epithelialization in the Dersani™ treated group in comparison to the control group treated with NaCl 0.9% and the group treated with 1% silver sulfadiazine (p<0.05) (Table 2; Fig. 2).

**Figure 3:**
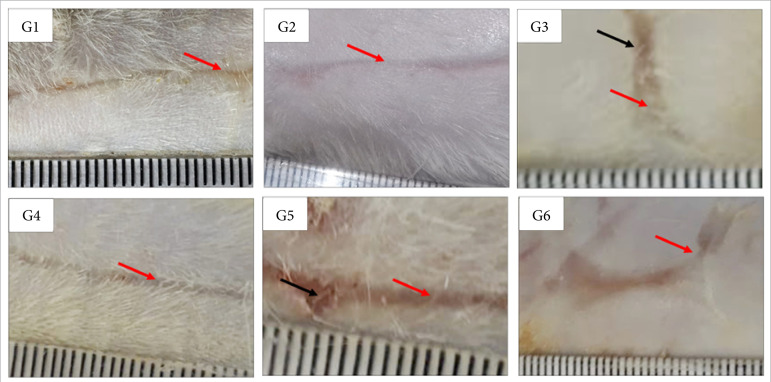
Macroscopic aspects of partial thickness burn wounds experimentally induced in Wistar rats 30 daysafter the injury induction. Scale in mm.

In the remodeling phase (30 DAI), there was greater necrosis/crust in the control group treated with NaCl 0.9% in comparison to the other treatments. Also, the Dersani™ treatment induced greater necrosis/crust than the Solosite™ one (p<0.05). There was less re-epithelialization in the Dersani™ treated group in comparison to the 1% silver sulfadiazine and the Debrigel™ treated group (p<0.05) (Table 2; Fig. 3).

**Table 2:** Macroscopic analysis of partial thickness burn wounds experimentally induced in Wistar rats treated with calcium and/or sodium alginate hydrogel-based dressings#.

Pathologic process	DAI	G1 (n=15) Median (min-max)	G2 (n=15) Median (min-max)	G3 (n=15) Median (min-max)	G4 (n=15) Median (min-max)	G5 (n=15) Median (min-max)	G6 (n=15) Median (min-max)	p-value	Dunn’s
Necrosis/crust	7	3 (2-3)	3 (2-3)	1 (0-2)	2 (2-3)	3 (2-3)	1 (1-2)	0.002*	G1>G3; G1>G6; G5>G3; G5>G6
	14	2 (1-3)	3 (2-3)	1 (0-1)	1 (0-1)	1 (1-2)	1 (0-1)	0.002*	G1>G3; G1>G4; G2>G3; G2>G4; G2>G5; G2>G6
	30	2.5 (2-3)	0 (0-1)	0 (0-1)	1 (0-1)	1 (1-2)	0 (0-1)	0.003*	G1>G2; G1>G3; G1>G4; G1>G6; G5>G6
Granulation tissue	7	2 (1-2)	1 (1-2)	1.5 (1-2)	3 (2-3)	1 (1-2)	3 (2-3)	0.003*	G4>G2; G4>G3; G4>G5; G6>G2; G6>G5
	14	2.5 (2-3)	2 (1-3)	2 (2-3)	2.5 (2-3)	3 (2-3)	3 (2-3)	0.512	
	30	0 (0-0)	0 (0-1)	0 (0-1)	0 (0-0)	0 (0-0)	0 (0-0)	0.202	
Re-epithelialization	7	0 (0-0)	0 (0-0)	0 (0-0)	0 (0-0)	0 (0-0)	0 (0-0)	1.00	
	14	0.5 (0-2)	0 (0-2)	2 (1-2)	2 (2-2)	2 (2-3)	1.5 (1-2)	0.014*	G5>G1; G5>G2
	30	3 (2-3)	3 (3-3)	3 (3-3)	3 (3-3)	2 (2-3)	3 (2-3)	0.013*	G2>G5; G3>G5

DAI: days after injury induction; n: number of animals per group; G1: control group with animals treated with NaCl 0.9%; G2: animals treated with 1% silver sulfadiazine; G3: animals treated with Debrigel™; G4: animals treated with Safgel™; G5: animals treated with Dersani Hydrogel™; G6: animals treated with Solosite™;

# results are expressed in median, minimum (min) and maximum (max) values. Statistical analysis: Kruskal-Wallis and Dunn’s post-test;

* statistical difference.

G1: control group with animals treated with NaCl 0.9%; G2: animals treated with 1% silver sulfadiazine; G3: animals treated with Debrigel™; G4: animals treated with Safgel™; G5: animals treated with Dersani Hydrogel™; G6: animals treated with Solosite™; black arrow: necrosis/crust; white arrow: granulation tissue.

G1: control group with animals treated with NaCl 0.9%; G2: animals treated with 1% silver sulfadiazine; G3: animals treated with Debrigel™; G4: animals treated with Safgel™; G5: animals treated with Dersani Hydrogel™; G6: animals treated with Solosite™; black arrow: necrosis/crust; white arrow: granulation tissue; red arrow: re-epithelialization.

G1: control group with animals treated with NaCl 0.9%; G2: animals treated with 1% silver sulfadiazine; G3: animals treated with Debrigel™; G4: animals treatedwith Safgel™; G5: animals treated with Dersani Hydrogel™; G6: animals treated with Solosite™; black arrow: necrosis/crust; red arrow: re-epithelialization.

### Microscopic analysis

**Figura 4:**
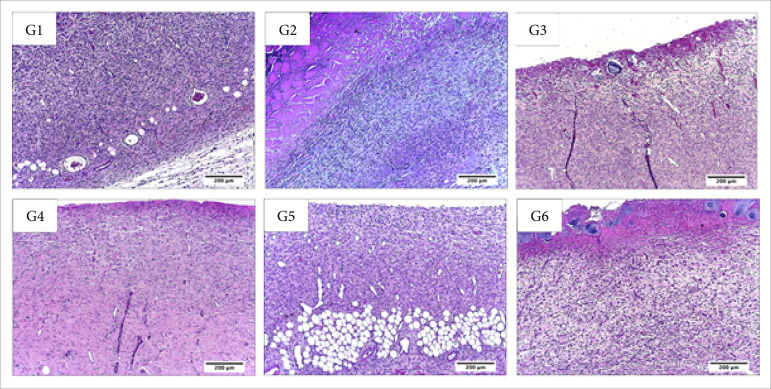
Microscopic aspects of partial thickness burn wounds experimentally induced in Wistar rats seven days after the injury induction. Stain hematoxylin and eosin. Augmentation: 10x scale in μm.

**Figura 5:**
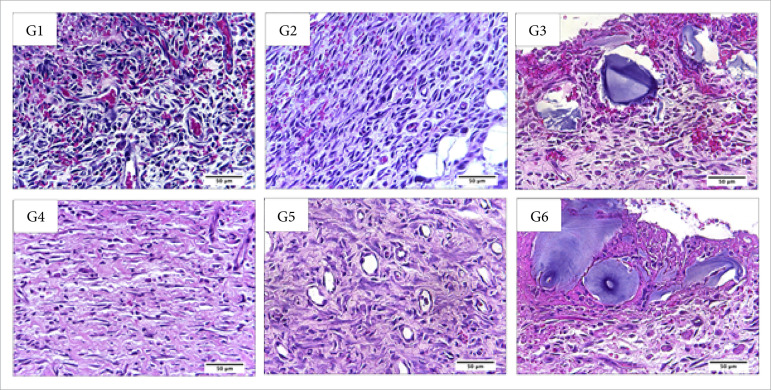
Microscopic aspects of partial thickness burn wounds experimentally induced in Wistar rats seven days after the injury induction. Stain hematoxylin and eosin. Augmentation: 40x scale in μm.

In the inflammatory phase of the healing process (seven DAI), there was less necrosis/crust in the wounds treated with Debrigel™ in comparison to the ones treated with NaCl 0.9% (control group) and with Solosite™ (p<0.05). The Solosite™ treated group presented more hemorrhage than the control one (p<0.05). The Debrigel™ and Solosite™ treated groups presented less fibrin than the one treated with 1% silver sulfadizine (p<0.05). Regarding the inflammatory infiltration, there was more PMN in the Solosite™ treated group in comparison to the Debrigel™ one; and there was more MN in the Solosite™ treated group in comparison to the 1% silver sulfadiazine and the Dersani™ treated ones (p<0.05) (Table 3, Figs. 4 and 5).

**Table 3:** Microscopic analysis of partial thickness burn wounds experimentally induced in Wistar rats treated with calcium and/or sodium alginate hydrogel-based dressings#.

Pathologic process	DAI	G1 (n=15) Median (min-max)	G2 (n=15) Median (min-max)	G3(n=15) Median (min-max))	G4 (n=15) Median (min-max)	G5 (n=15) Median (min-max)	G6 (n=15) Median (min-max)	p-value	Dunn’s
Necrosis/crust	7	2 (1-3)	1 (1-2)	0 (0-1)	1 (1-2)	1.5 (1-2)	1 (1-3)	0.041*	G1>G3; G6>G3
	14	1 (1-1)	2 (1-3)	1 (1-1)	1.5 (1-2)	1 (0-2)	0 (0-1)	0.021*	G2>G6; G4>G6
	30	0 (0-1)	1 (0-1)	0.5 (0-1)	0 (0-1)	1 (0-2)	0 (0-0)	0.134	
Hemorrhage	7	1.5 (0-2)	2 (1-2)	2 (0-3)	1 (1-2)	2.5 (2-3)	3 (2-3)	0.010*	G6>G1
	14	0 (0-0)	1 (1-2)	1 (0-2)	1 (0-1)	1 (0-2)	1.5 (0-2)	0.033*	G6>G1
	30	0 (0-0)	0 (0-0)	0 (-0-0)	0 (0-0)	0 (0-0)	0 (0-0)	1.000	
Fibrin	7	1 (1-2)	2 (2-2)	0 (0-2)	1 (1-2)	1 (1-2)	1 (0-2)	0.013*	G2>G3; G2>G6
	14	1 (0-1)	1 (1-2)	1 (0-3)	1 (0-2)	0 (0-1)	0 (0-1)	0.100	
	30	0 (0-0)	0 (0-0)	0 (0-0)	0 (0-0)	0 (0-0)	0 (0-0)	1.000	
PMN	7	1.5 (1-2)	1 (1-2)	0 (0-1)	1 (1-2)	1.5 (1-2)	1 (1-3)	0.005*	G6>G3
	14	1 (1-1)	1.5 (1-2)	1 (1-1)	2 (2-2)	1 (1-3)	0 (-01)	0.005*	G2>G6; G4>G6; G5>G6
	30	0 (0-1)	1 (0-1)	0 (0-1)	0 (0-0)	1 (0-1)	0 (0-1)	0.063	
MN	7	2 (2-3)	3 (3-3)	2 (1-3)	3 (2-3)	3 (3-3)	2 (2-2-)	0.005*	G2>G6; G5>G6
	14	2 (2-3)	2 (2-2)	2 (1-3)	2.5 (1-3)	3 (2-3)	2 (2-3)	0.534	
	30	1 (1-2)	2 (1-2)	1 (1-2)	1 (1-2)	1.5 (1-2)	1 (1-1)	0.321*	
Angiogenesis	7	3 (3-3)	3 (3-3)	3 (3-3)	3 (3-3)	3 (3-3)	3 (3-3)	1.000	
	14	3 (2-3)	2 (2-2)	3 (2-3)	3 (3-3)	3 (2-3)	3 (2-3)	0.012*	G1>G2; G4>G2; G6>G2
	30	1 (1-1)	1 (1-2)	1 (1-1)	1.5 (1-2)	2.5 (1-3)	1 (1-1)	0.040*	G5>G1; G5>G3; G5>G6
Fibroblast	7	3 (3-3)	3 (3-3)	3 (3-3)	3 (3-3)	3 (3-3)	3 (3-3)	1.000	
	14	3 (3-3)	3 (3-3)	2.5 (2-3)	3 (3-3)	3 (2-3)	3 (2-3)	0.175	
	30	2 (2-3)	2 (2-2)	2 (2-2)	2 (1-2)	2,5 (2-3)	1 (1-2)	0,022*	G1>G6; G5>G6

DAI: days after injury induction; n: number of animals per group; G1: control group with animals treated with NaCl 0.9%; G2: animals treated with 1% silver sulfadiazine; G3: animals treated with Debrigel™; G4: animals treated with Safgel™; G5: animals treated with Dersani Hydrogel™; G6: animals treated with Solosite™; PMN: polymorphonuclear cells infiltration; MN: mononuclear cells infiltration;

# results are expressed in median, minimum (min) and maximum (max) values. Statistical analysis: Kruskal-Wallis and Dunn’s post-test;

* statistical difference.

G1: control group with animals treated with NaCl 0.9%; G2: animals treated with 1% silver sulfadiazine; G3: animals treated with Debrigel™; G4: animals treated with Safgel™; G5: animals treated with Dersani Hydrogel™; G6: animals treated with Solosite™.

G1: control group with animals treated with NaCl 0.9%; G2: animals treated with 1% silver sulfadiazine; G3: animals treated with Debrigel™; G4: animals treated with Safgel™; G5: animals treated with Dersani Hydrogel™; G6: animals treated with Solosite™.

**Figura 6:**
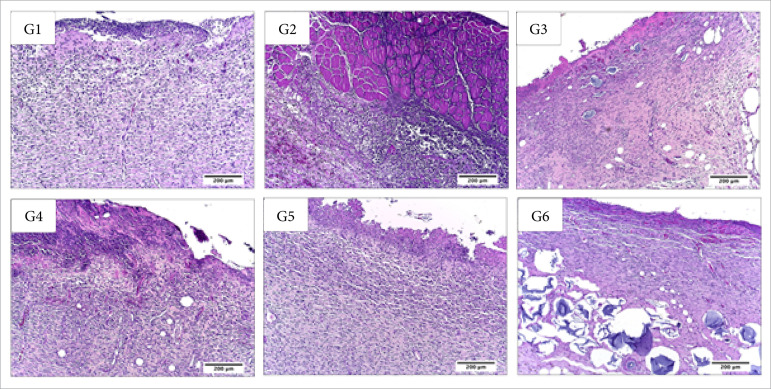
Microscopic aspects of partial thickness burn wounds experimentally induced in Wistar rats 14 days after the injury induction. Stain hematoxylin and eosin. Augmentation: 10x scale in μm.

**Figura 7:**
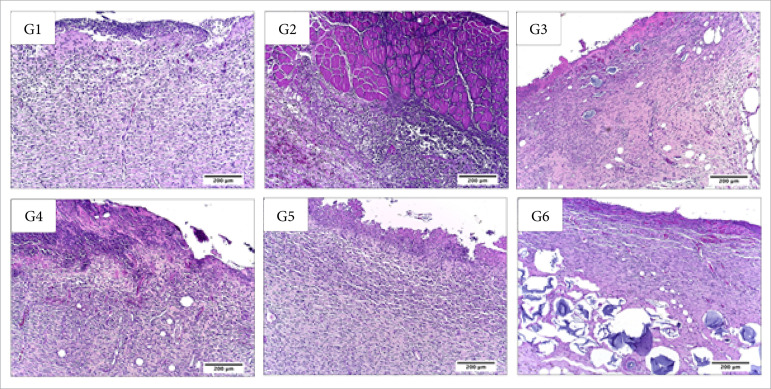
Microscopic aspects of partial thickness burn wounds experimentally induced in Wistar rats 14 days after the injury induction. Stain hematoxylin and eosin. Augmentation: 40x scale in μm.

In the proliferative phase of the healing process, there was less necrosis/crust in the Solosite™ treated group in comparison to the 1% silver sulfadiazine and Safgel™ treated ones (p<0.05). The Solosite™ treated group presented more hemorrhage than the control one (p<0.05). There was less PMN inflammatory infiltration in the Solosite™ treated group in comparison to the 1% silver sulfadiazine, Safgel™ and Dersani™ treated ones (p<0.05). There was less angiogenesis in the 1% silver sulfadiazine treated group in comparison to the NaCl 0.9%, Safgel™ and Solosite™ treated ones (p<0.05) (Table 3, Figs. 6 and 7).

G1: control group with animals treated with NaCl 0.9%; G2: animals treated with 1% silver sulfadiazine; G3: animals treated with Debrigel™; G4: animalstreated with Safgel™; G5: animals treated with Dersani Hydrogel™; G6: animals treated with Solosite™.

G1: control group with animals treated with NaCl 0.9%; G2: animals treated with 1% silver sulfadiazine; G3: animals treated with Debrigel™; G4: animalstreated with Safgel™; G5: animals treated with Dersani Hydrogel™; G6: animals treated with Solosite™.

**Figura 8:**
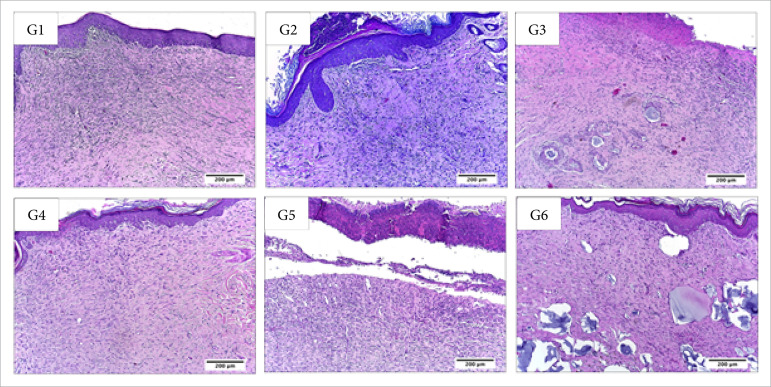
Microscopic aspects of partial thickness burn wounds experimentally induced in Wistar rats 30 daysafter the injury induction. Stain hematoxylin and eosin. Augmentation: 10x scale in μm.

**Figura 9:**
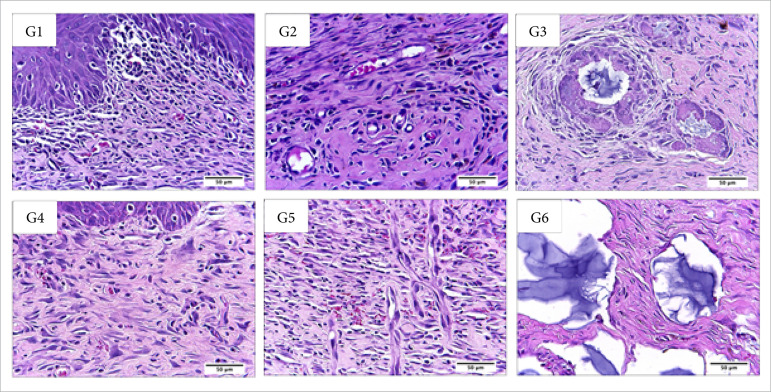
Microscopic aspects of partial thickness burn wounds experimentally induced in Wistar rats 30 daysafter the injury induction. Stain hematoxylin and eosin. Augmentation: 40x scale in μm.

In the remodeling phase of the healing process, there was less fibroblasts in the Solosite™ treated group in comparison to the NaCl 0.9% and Dersani™ treated ones (p<0.05) (Table 3, Figs. 8 and 9).

G1: control group with animals treated with NaCl 0.9%; G2: animals treated with 1% silver sulfadiazine; G3: animals treated with Debrigel™; G4: animals treated with Safgel™; G5: animals treated with Dersani Hydrogel™; G6: animals treated with Solosite™.

G1: control group with animals treated with NaCl 0.9%; G2: animals treated with 1% silver sulfadiazine; G3: animals treated with Debrigel™; G4: animals treated with Safgel™; G5: animals treated with Dersani Hydrogel™; G6: animals treated with Solosite™.

Moreover, the Debrigel™ and Solosite™ treatments induced an impregnation of a basophilic material in the epithelial and dermic layers of the skin with an intensity that varied from discrete to accentuated. Surrounding this impregnation, there was an inflammatory response that evolved to the formation of a granuloma.

The treatments used did not interfere in the time for the wound closure (Table 4). However, the Solosite™ treated group presented microorganisms in the wound at seven DAI, which was not observed in any other treatment (Table 5).

**Table 4:** Wound closure of partial thickness burn wounds experimentally induced in Wistar rats treated with calcium and/or sodium alginate hydrogel-based dressings*.

Wound closure – 30 DAI	G1	G2	G3	G4	G5	G6	p-value
Yes	4	4	4	4	1	3	1.00
No	1	1	1	1	4	2	
Total	5	5	5	5	5	5	

DAI: days after injury induction; G1: control group with animals treated with NaCl 0.9%; G2: animals treated with 1% silver sulfadiazine; G3: animals treated with Debrigel™; G4: animals treated with Safgel™; G5: animals treated with Dersani Hydrogel™; G6: animals treated with Solosite™;

* statistical analysis: Fisher’s exact test.

**Table 5:** Microscopic detection of microorganisms in partial thickness burn wounds experimentally induced in Wistar rats treated with calcium and/or sodium alginate hydrogel-based dressings*.

Microorganisms presence – 7 DAI	G1	G2	G3	G4	G5	G6	p-value
Yes	0	0	0	0	0	3	1.00
No	5	5	5	5	5	2	
Total	5	5	5	5	5	5	

DAI: days after injury induction; G1: control group with animals treated with NaCl 0.9%; G2: animals treated with 1% silver sulfadiazine; G3: animals treated with Debrigel™; G4: animals treated with Safgel™; G5: animals treated with Dersani Hydrogel™; G6: animals treated with Solosite™;

* statistical analysis: Fisher’s exact test.

### Collagen fibers quantification

**Figure 10:**
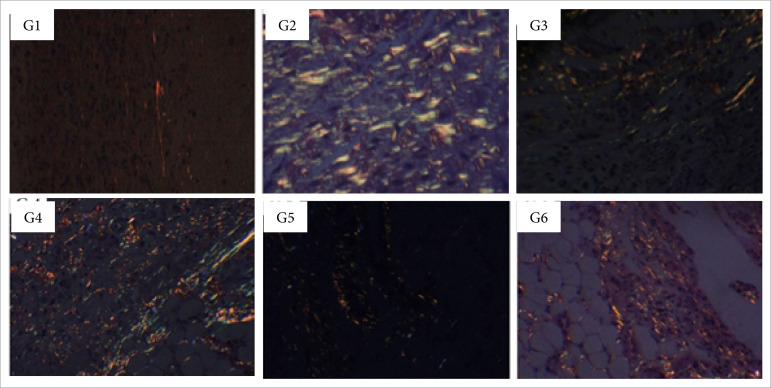
Collagen fibers deposition in partial thickness burn wounds experimentally induced in Wistar rats seven days after the injury induction (DAI). Collagen fibers type I are shown in red and type III in green.Stain Picrosirius red. Augmentation: 20x scale in μm.

In the inflammatory phase of the healing process (seven DAI), all treatments induced more collagen fibers deposition in comparison to the control group, treated with NaCl 0.9%, with the exception of the Solosite™ treatment (p<0.05). The Debrigel™ and Dersani™ treatments induced more collagen fibers than the 1% silver sulfadiazine (p<0.05). The Safgel™ and Solosite™ treatments induced greater deposition of collagen fibers than the Debrigel™ one, and the Safgel™ treatment induced less deposition than the Solosite™ one (p<0.05) (Table 6, Fig. 10).

**Table 6:** Quantitative analysis of collagen fibers in partial thickness burn wounds experimentally induced in Wistar rats treated with calcium and/or sodium alginate hydrogel-based dressings#.

DAI	G1 (n=15) Median (min-max)	G2 (n=15) Median (min-max)	G3 (n=15) Median (min-max)	G4 (n=15) Median (min-max)	G5 (n=15) Median (min-max)	G6 (n=15) Median (min-max)	p-value	Dunn’s
7	0.03 (0.00-1.51)	0.74 (0.00-15.03)	0.28 (0.00-3.72)	1.49 (0.02-16.03)	0.20 (0.01-3.87)	0.72 (0.00-17.13)	0.001*	G2>G1; G3>G1; G4>G1; G5>G1; G3>G2; G5>G2; G4>G3; G6>G3; G6>G4
14	0.02 (0.01-5.02)	1.79 (0.02-15.62)	3.20 (0.02-17.31)	2.13 (0.09-23.32)	3.21 (0.02-19.82)	1.67 (0.03-17.59)	0.001*	G2>G1; G3>G1; G4>G1; G5>G1; G6>G1; G6>G3; G6>G5
30	0.02 (0.00-4.25)	4.26 (0.12-18.73)	9.97 (0.13-38.27)	7.47 (0.25-30.13)	8.04 (0.71-31.70)	6.30 (0.04-20.45)	0.001*	G2>G1; G3>G1; G4>G1; G5>G1; G6>G1

DAI: days after injury induction; n: number of animals per group; G1: control group with animals treated with NaCl 0.9%; G2: animals treated with 1% silver sulfadiazine; G3: animals treated with Debrigel™; G4: animals treated with Safgel™; G5: animals treated with Dersani Hydrogel™; G6: animals treated with Solosite™;

# results expressed in median (minimum-maximum). Statistical analysis: Kruskal-Wallis and Dunn’s post-test;

* statistical difference.

G1: control group with animals treated with NaCl 0.9%; G2: animals treated with 1% silver sulfadiazine; G3: animals treated with Debrigel™; G4: animals treated with Safgel™; G5: animals treated with Dersani Hydrogel™; G6: animals treated with Solosite™.

**Figure 11:**
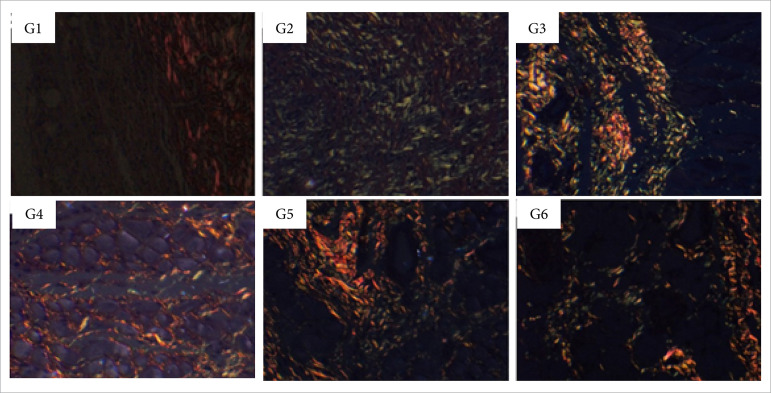
Collagen fibers deposition in partial thickness burn wounds experimentally induced in Wistar rats 14 days after the injury induction (DAI). Collagen fibers type I are shown in red and type III in green.Stain Picrosirius red. Augmentation: 20x scale in μm.

In the proliferative phase (14 DAI), all the treatments induced greater collagen fibers deposition than the control group, treated with NaCl 0.9% (p<0.05). Also, the Solosite™ treatment induced greater deposition than the Debrigel™ and Dersani™ ones (p<0.05) (Table 6, Fig. 11).

G1: control group with animals treated with NaCl 0.9%; G2: animals treated with 1% silver sulfadiazine; G3: animals treated with Debrigel™; G4: animals treated with Safgel™; G5: animals treated with Dersani Hydrogel™; G6: animals treated with Solosite™.

**Figure 12:**
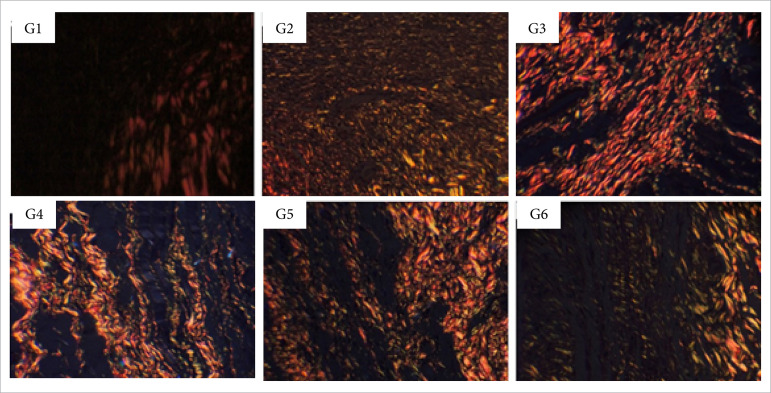
Collagen fibers deposition in partial thickness burn wounds experimentally induced in Wistar rats 30 days after the injury induction (DAI). Collagen fibers type I are shown in red and type III in green. Stain Picrosirius red. Augmentation: 20x scale in μm.

While in the remodeling phase (30 DAI), all treatments induced more collagen fibers deposition than the control group, treated with NaCl 0.9% (p<0.05) (Table 6, Fig. 12).

G1: control group with animals treated with NaCl 0.9%; G2: animals treated with 1% silver sulfadiazine; G3: animals treated with Debrigel™; G4: animals treated with Safgel™; G5: animals treated with Dersani Hydrogel™; G6: animals treated with Solosite™.

## Discussion

This study compared several calcium and/or sodium alginate hydrogel-based dressings in the treatment of partial thickness burn wounds experimentally induced in Wistar rats and followed through a 30-day period, in order to determine which treatment is better in each phase of the healing process. It is important to emphasize that this is an experimental study, and our findings regard the animal’s response to the treatments performed during the experimental period of 30 days.

It is interesting to highlight that the treatment with Dersani™ induced better results during the inflammatory and proliferative phases of the healing process. The animals treated with Safgel™ presented better scaring in the remodeling phase, and the treatments with Dersani™ and Solosite™ induced greater wound closure.

Hydrogel-based dressings may be used as first aid in burn wounds management as an alternative to quickly cooling the wound in cases of lack of clean water, hypothermia, or large extent burns. These dressings are important to protect the wound from contamination and reduce pain^19^. The maintenance of moist and humidity in the burn wound bed is essential to hydrate the wound, absorb the exudate and induce autolysis of the necrotic tissue^18^^,^ 25.

According to the literature and the manufacturer, Debrigel™ has calcium and sodium alginate in its composition, which when in contact with the exudate forms a hydrophilic and non-adherent gel that provides a moist environment on the burn bed, promoting autolytic debridement and absorbing the excess exudate. This property allows trauma-free removal, with little or no damage to the tissue, with less formation of necrosis in the wound bed. It also has sodium carboxymethyl cellulose, which stimulates angiogenesis and autolytic debridement and accelerates the tissue granulation process^19^^,^ 26 ^-^ 28.

On the other hand, the propylene glycol presents in Debrigel™ formulation removes necrotic tissue through autolytic debridement and stimulates the release of exudate^29^^-^ 32. While other substances such as potassium sorbate, hydantoin and benzyl alcohol present in Debrigel™ composition have fungicides, bactericide, and antiseptic action on the wound bed^33^. Also, triethanolamine has healing potential, aiding angiogenesis in the first phase of healing, as described by Zhou et al.^34^ in a study which formulated a gel with triethanolamine which promoted the cutaneous repair in surgical wounds experimentally induced in mice.

According to other studies and to the manufacturer, Solosite™ has allantoin in its formulation, which has a stimulating action on cell proliferation. This substance is hydrolyzed in the skin to form urea, which has a moisturizing and keratolytic action and activates the wound healing^35^. In a study that compared the effect of allantoin in experimental wounds performed in Chinchilla rabbits, it was found that this product has an angiogenic and healing action^33^. In another study using Wistar rats, the authors showed that allantoin is responsible for the healing and astringent effect, stimulating the formation of granulation tissue^36^^,^ 37. Glycerin, also present in the Solosite™ formulation, has chemotactic potential for leukocytes, favors angiogenesis, promotes autolytic debridement, keeps the burn medium moist, that is, ideal for preventing necrosis and favoring the formation of granulation tissue^38^. The presence of allantoin in these hydrogel-based dressings is very positive since it has no irritating effect when applied to the skin, as it binds to the stratum corneum, increasing the affinity of keratin with water, allowing hydration in the wound bed, and facilitating debridement of the wound which contributes to the healing process^36^^,^ 37 ^,^ 39 ^,^ 40.

Sodium carboxymethyl cellulose, present in Debrigel™, Safgel™ and Solosite™ products, has the function of performing ion exchange, making the burn bed hydrated, thus reducing the formation of necrosis/crust, while methylparaben and propylparaben have antimicrobial action^41^. It is proposed that paraben acts on the synthesis of DNA and RNA, on ATPases and phosphotransferases, and even on the mechanisms of transport through membranes acting on angiogenesis and cell proliferation, which contributes to all phases of the healing process^41^.

Debrigel™ and Solosite™ products are believed to be the most recommended in the inflammatory phase due to the possibility of hydrating the wound bed, favoring autolytic debridement, preventing the formation and/or facilitating the removal of necrosis/crust^15^. Other authors who used porcine models for burn wound studies showed that treatment with Debrigel™ and Solosite™ products decreased burn depth and reduced the number of bacteria more efficiently than silver sulfadiazine cream^42^.

When analyzing the different treatments in the proliferative phase, it was observed that the lesions treated with silver sulfadiazine had lower angiogenesis than those treated with Safgel™, Solosite™, and the control. In the remodeling phase, it was observed that, with the use of Dersani™, there was greater angiogenesis when compared to the control and the products Debrigel™ and Solosite™. While Solosite™ treatment induced less fibroblasts than the control and Dersani™, Safgel™ has in its formulation compounds that have the potential to leave the wound bed moist, controlling exudate and favoring ion exchange, oxygenation, and nutrients. It is believed that this effect provided better granulation tissue than Debrigel™ and Solosite™^13^^,^ 16 ^,^ 24.

Some studies describe that, after adding hydrogel, silver sulfadiazine provided greater angiogenesis and re-epithelialization in comparison to silver sulfadiazine used alone^18^^,^ 19 ^,^ 40 ^,^ 43. In our study, in general, there was a benefit when using the hydrogel instead of silver sulfadiazine, improving the healing process.

In the proliferative phase, burns treated with Safgel™ and Solosite™ showed greater angiogenesis than the group treated with silver sulfadiazine. It is reported in the literature that wounds treated with Safgel™ and Solosite™ presented greater chemotaxis of cells to the wound bed, increased angiogenesis, and kept the environment moist, in addition to accelerating the granulation tissue process^44^^,^ 45. Other study showed that the application of Safgel™ and Solosite™ products had greater absorption, forming a protective film on the lesion and skin, and providing increased local cellular nutrition, improving angiogenesis^46^.

The use of Dersani™ during the remodeling phase of the healing process induced a greater number of fibroblasts. A study by Zhang et al.^47^ showed that this product had the ability to migrate and increase the number of fibroblasts, being able to induce the migration of cells to the wound bed and consequently improve the wound closure. Thus, fibroblasts grow in number and form a new provisional extracellular matrix through the secretion of collagen and fibronectin^47^. Bainbridge^48^ has correlated positively the presence of fibroblasts to angiogenesis. In addition, fibroblasts are potent modulators of cell proliferation, motility and differentiation, which are important factors that contribute to the success of the healing process^49^. It is therefore believed that fibroblasts play a central role in the initiation of angiogenesis. Solosite™, which is a cross-linked carboxymethylcellulose-based hydrogel, had the ability to keep the wound bed moist, favoring the presence of fibroblasts and angiogenesis^45^.

In the present study, it was possible to observe that Dersani™ continued to induce angiogenesis up to 30 days after lesion induction. This finding may not be beneficial to the lesion in the remodeling phase, thus restricting the use of the product until the proliferative phase. Lesions treated with Safgel™ and Solosite™ showed greater formation of granulation tissue and angiogenesis in the present study, showing that hydrogel-based dressings are capable to accelerate angiogenesis and collagen deposition in burn wounds^50^.

When analyzing the wounds treated with Solosite™, a greater amount of PMN was observed in comparison to the ones treated with Debrigel™. It is believed that the greater number of PMN cells at the inflammatory phase of the healing process may be due to increased necrosis/crust in burns treated with Solosite™, thus amplifying the inflammatory process^13^^,^ 16. Burns treated with silver sulfadiazine and Dersani™ had a higher number of MN cells than lesions treated with Solosite™ at the proliferative phase of the healing process. This situation may increase the number of macrophages in the wound bed, thus causing phagocytosis of cell debris^46^^,^ 51 ^,^ 52.

The lesions treated with Solosite™ during the inflammatory phase of the healing process showed greater hemorrhage than with the use of the control, possibly due to the mechanical trauma caused by cleaning the wound bed. Other authors described that necrosis, hemorrhage, hyperemia, and fibrin are common processes in the period of hemostasis, inflammation and migration of cells to the injured region, such as platelets, neutrophils, lymphocytes, endothelial cells, and, later, macrophages, aiding in angiogenesis^53^^,^ 54. While in the proliferative phase, it was verified that the lesions treated with Solosite™ continued to present greater hemorrhage than the lesions treated with the NaCl 0.9% control; it is believed that this occurred because of the injury handling during debridement and dressing changes that could have caused trauma, which is in accordance to other studies^55^^,^ 56.

Dersani™ can induce re-epithelialization faster than both controls, as demonstrated in the results, thus contributing positively to the healing process. According to the literature, the high-water content in its composition favors skin hydration and re-epithelialization^52^^,^ 57. In another study, Dersani™ was used together with calcium alginate and collagenase to treat surgical injury for 14 days, and after this period only Dersani™ was used, resulting in better re-epithelialization of the lesion^58^.

During the remodeling phase, it was possible to verify that with the use of silver sulfadiazine and Debrigel™ there was greater re-epithelialization of the lesions than with the use of Dersani™. Silver sulfadiazine kept the wound bed in favorable conditions for the formation of granulation tissue^52^, while the wounds treated with Dersani™ continued to present necrosis/crust. This process may justify a delay in the process of re-epithelialization and wound closure.

Regarding the collagen quantification, in the inflammatory phase, the treatment of the wounds should induce greater production of type III collagen. This type of collagen is commonly found in soft tissues, such as blood vessels, dermis and fascia, whereas granulation tissue has the potential to express 30 to 40% of type III collagen, which is considered a more immature collagen tissue, i.e., found at the beginning of the healing process^18^^,^ 24 ^,^ 33. The hydrogel-based dressings had the potential to present and activate mechanisms that induced the production of type III collagen fibers^18^^,^ 24 ^,^ 33. Thus, at this phase of the healing process, the products with hydrogel induced more collagen fibers than the NaCl 0.9% and silver sulfadiazine control groups.

In the proliferative phase, all products were able to induce more collagen fibers than the NaCl 0.9% control group. In this phase, collagen degradation begins being mediated by specific collagenases. Thus, type III collagen begins to give way to type I collagen. This type of collagen has thicker and more resistant fibers, in which it will predominate until the end of the remodeling phase^53^^,^ 54. It is worth noticing that all products showed a greater expression of collagen fibers than the control, thus inducing the phase of replacement of type III collagen for type I collagen. This reinforces the importance of applying a treatment in burn wounds in addition to the cleaning with saline solution. This way, the products can enhance the progress to the remodeling phase by replacing type III collagen fibers by type I ones.

In the remodeling phase, the healing process is responsible for increasing the resistance of the wound bed. At the end of the inflammatory phase, 3% of the intact skin’s resistance is restored; at the end of the remodeling phase about 30%; and after about three months, 80%. Thus, in the remodeling phase, there was a decrease in collagen deposition, and the change from type III to type I collagen fiber^59^. In our study, wounds treated with all products had a greater amount of collagen fibers than those treated with the NaCl 0.9% control.

## Conclusions

Regarding the results obtained in the experimental model of burn wounds in rats, it was possible to conclude that the use of hydrogels-based products in the treatment of partial thickness burn wounds experimentally induced in rats provided a better effect in the healing process than the use of conventional treatments such as silver sulfadiazine and NaCl 0.9%, that is, it decreased necrosis, increased granulation tissue formation, and re-epithelialization. Also, the hydrogel-based products induced greater collagen fibers production and maturation, showing beneficial effect when used in the treatment of burn wounds.
